# Pre-hypertension and the risk of diabetes mellitus incidence using a marginal structural model in an Iranian prospective cohort study

**DOI:** 10.4178/epih.e2018026

**Published:** 2018-06-23

**Authors:** Ahmad Khosravi, Mohammad Hassan Emamian, Hassan Hashemi, Akbar Fotouhi

**Affiliations:** 1 Center for Health Related Social and Behavioral Sciences Research, Shahroud University of Medical Sciences, Shahroud, Iran; 2 Department of Epidemiology, School of Public Health, Shahroud University of Medical Sciences, Shahroud, Iran; 3 Noor Ophthalmology Research Center, Noor Eye Hospital, Tehran, Iran; 4 Department of Epidemiology and Biostatistics, School of Public Health, Tehran University of Medical Sciences, Tehran, Iran

**Keywords:** Blood pressure, Causal inference, Diabetes mellitus, Inverse probability weight, Prehypertension, Iran

## Abstract

**OBJECTIVES:**

The aim of this study was to evaluate the effect of pre-hypertension and its sub-classification on the development of diabetes.

**METHODS:**

In this cohort study, 2,941 people 40 to 64 years old without hypertension or diabetes were followed from 2009 through 2014. According to the Joint National Committee on Prevention, Detection, Evaluation, and Treatment of High Blood Pressure (JNC)-7 criteria, we classified participants into normal and pre-hypertension groups. The effect of pre-hypertension on the 5-year incidence rate of diabetes was studied using inverse probability of treatment weighting. We modeled the exposure and censored cases given confounding factors such as age, sex, body mass index, smoking, economic status, and education.

**RESULTS:**

The 5-year incidence rate of diabetes among people with pre-hypertension and those with normal blood pressure (BP) was 12.7 and 9.7%, respectively. The risk ratio (RR) for people with pre-hypertension was estimated to be 1.13 (95% confidence interval [CI], 0.90 to 1.41). The RRs among people with normal BP and high-normal BP, according to the JNC-6 criteria, compared to those with optimal BP were 0.96 (95% CI, 0.73 to 1.25) and 1.31 (95% CI, 1.01 to 1.72), respectively.

**CONCLUSIONS:**

Our results showed that participants who had higher levels of BP (high-normal compared to optimal BP) had a higher risk of diabetes development. With regard to the quantitative nature of BP, using the specifically distinguishing of stage 1 hypertension or high-normal BP may be a more meaningful categorization for diabetes risk assessment than the JNC-7 classification.

## INTRODUCTION

Hypertension is a risk factor for cardiovascular disease (CVD) [1] and diabetes [2,3]. High systolic blood pressure (SBP; > 115 mmHg) is associated with an increased risk of CVDs [4]. According to definition of seventh report of the Joint National Committee on Prevention, Detection, Evaluation, and Treatment of High Blood Pressure (JNC)-7, pre-hypertension is a SBP between 120 and 139 mmHg and/or a diastolic blood pressure (DBP) between 80 and 89 mmHg [5], and pre-hypertension is associated with an increased risk of CVDs [6,7]. Currently, the prevalence of diabetes as a major cause of morbidity and mortality [8] is increasing in most countries [9]. Diabetes is also associated with a high risk of cardiovascular mortality and morbidity [10]. The relationship of heart disease with diabetes and hypertension has been investigated in several studies [11]. Various studies have also shown that diabetes and hypertension are associated with obesity [12,13]. Biologically, due to the relationship of hypertension with chronic inflammation and endothelial dysfunction and their role in increasing the risk of diabetes, it appears that there is a relationship between diabetes and hypertension [14,15]. Several studies have suggested that there is a significant relationship between hypertension and the risk of developing diabetes [2,3,16], but controversial results have been reported on the relationship between prehypertension and diabetes. Cross-sectional studies have shown no significant relationship between diabetes and pre-hypertension [13,17,18]. A longitudinal study conducted on 2,768 non-diabetic subjects with normal blood pressure (BP) showed that after adjusting for body mass index (BMI), pre-hypertension was not associated with an increased risk of developing diabetes [19]. According to the sixth edition of the categorization presented by the JNC-6, people with pre-hypertension were divided into 2 groups: those with high-normal BP (130-139/85-89 mmHg) and those with normal BP (120-129/80-84 mmHg), while those with SBP below 120 mmHg and DBP below 80 mmHg were regarded as having optimal BP [20]. In a new guideline published by the American College of Cardiology (ACC) and the American Heart Association (AHA) on hypertension, a new BP classification system similar to the JNC-6 was released. In this new guideline, the category of pre-hypertension was deleted, and individuals with prehypertension were divided into the categories of elevated BP (120- 129 and < 80 mmHg) and stage 1 hypertension, defined as a SBP of 130-139 mmHg or a DBP of 80-89 mmHg [21]. Additionally, the International Diabetes Federation released a uniform definition of metabolic syndrome that is clinically applicable worldwide, with a set of criteria including BP over 130/85 mmHg [22]. Therefore, this BP range may be more important for assessing the risk of diabetes than the JNC-7 category of pre-hypertension.

In a 10-year cohort study, the risk of diabetes in females with high-normal BP and females with hypertension was reported to be 1.45 and 2.03 times higher, respectively, than that of females with normal BP [2]. In other cohort studies, an increase in BP to levels above normal was associated with an increase in the hazard of diabetes [3,23]. Given the controversial results obtained from other studies about the relationship between pre-hypertension and diabetes, this study used data from a prospective cohort study and was conducted using a causal approach to answer the following questions: Is pre-hypertension a risk factor for type 2 diabetes? Is it necessary to divide pre-hypertension into 2 stages?

## MATERIALS AND METHODS

### Data

To answer these research questions, data from the Shahroud Eye Cohort Study (ShECS) were used. The ShECS was conducted in 2 phases in 2009 and 2014. The details of the methodology of the study have been presented elsewhere [24]. A total of 5,190 people from the 40- to 64-year-old population of Shahroud (northeast Iran) participated in the first phase. After 5 years, in 2014 the same people were interviewed and examined again in the second phase. A total of 453 people (7.8%) did not participate in the second phase of the study. Further investigation revealed that 108 people had died within the 5-year interval. The study protocol was reviewed and approved by the ethics committee of Shahroud University of Medical Sciences at both phases. After obtaining written consent from each participant, he or she was interviewed and clinical examinations were performed.

For this study, 2,941 of the participants in the first phase who had not been diagnosed with hypertension or diabetes were selected, and based on their BP in the first phase of the study, they were classified into pre-hypertension and normal BP groups, according to the JNC-7 criteria [5]. The role of pre-hypertension in development of diabetes within a 5-year period was studied using inverse probability treatment weighting (IPTW). For comparison, in another analysis, we divided people with pre-hypertension into those with high-normal BP and those with normal BP according to the JNC-6 and ACC criteria [20,21]. We assessed the relationships of these categorizations with diabetes incidence, compared with participants with a SBP below 120 mmHg and a DBP below 80 mmHg, which was regarded as optimal BP.

### Measurement

Details of the BP, BMI [13], and diabetes [16] measurements in the second phase of the study have been presented elsewhere. BP was measured on the right arm, by a trained nurse. BP was measured twice on the same day with an interval of 3 minutes. In this study, individuals with fasting blood glucose level ≥ 126 mg/dL and/or those with a hemoglobin A1c level ≥ 6.5% (48 mmol/mol) were considered to have diabetes, as were individuals who had been diagnosed with diabetes and used medications to lower their blood sugar [16,25]. Modeling of the exposure was done based on age, sex, marital status, economic status (with levels of low, medium, and high), education, smoking, and BMI.

### Analysis

In observational studies, since the exposure is not made by the researcher (there is no intervention) and it is not assigned randomly to the participants, it is difficult to assess the causal effects of exposure [26]. Furthermore, the probability of exposure can be influenced by extraneous factors, or confounders, which can also affect the outcome of the study. Therefore, the causal effect of the exposure is also influenced by confounding factors. In observational studies, such as cohort studies, that examine the effect of the exposure on the outcome, the usual statistical approach is to use multiple regression models. Marginal structural models, including IPTW, are an alternative to regression models. IPTW is based on a counterfactual framework. Its aim is to create a pseudo-population in which the associations between confounder variables and the exposure have been eliminated, resulting in independency between the exposure and confounders in the pseudopopulation [27]. In this method, each individual’s weight is estimated based on the probability of exposure, given the confounders present, and the resulting weight is called the propensity score (PS = Pr [exposure = 1| confounders]) [26,28]. This conditional probability of exposure was estimated based on logistic regression models using BMI, age (restricted cubic splines with 3 knots), and education as continuous variables and marital status, sex, economic status, and smoking as categorical variables.

To stabilize the inverse probability (IP) treatment weight, the numerator of the fraction weight for people with pre-hypertension (exposed) was set as the probability of pre-hypertension and the numerator of the fraction weight for people with normal BP (unexposed) was set as 1 minus the probability of pre-hypertension (stabilized inverse probability treatment weight: Pr [exposure = 1])/Pr [exposure = 1| confounders] for pre-hypertension and 1- Pr [exposure= 1]/1-Pr [exposure= 1|confounders] for individuals with normal BP) [27].

We ran a multinomial logit model to estimate propensity scores for each category of BP (optimal, normal, and high-normal).

Of the 2,941 participants, 295 had an unknown diabetes status in the second phase of the study, including those who died in the follow-up interval and those who left the study. To adjust for selection bias due to censoring, the censoring of the participants was modeled based on the variables in the first phase of the study, including age, sex, marital status, smoking, BMI, socioeconomic status, and education. Finally, the stabilized IP weight of censoring as the weight of censorship in the study multiplied by the stabilized IP weight of exposure was entered into the model as the stabilizing IP weight, and the weighted average of diabetes incidence for the entire population and for the pre-hypertension group was estimated in a counterfactual model. Based on this, the average treatment effect (ATE) was calculated [26,29]. To estimate the risk ratio (RR), a generalized linear model with binomial family and log link was used. The cluster design effect was considered to calculate the confidence interval and to estimate the standard error. Data analysis was performed using Stata version 12 (StataCorp., College Station, TX, USA) and the significance level was set at 0.05.

### Ethics statements

The study protocol was approved by the institutional review board (IRB) of Shahroud University of Medical Sciences, Shahroud, Iran (930/09). Informed consent was confirmed by the IRB.

All procedures performed in this study involving human participants were conducted in accordance with the ethical standards of the Shahroud University Ethics Review Committee and with the 1964 Declaration of Helsinki and its later amendments.

## RESULTS

The demographic and baseline characteristics of the participants with normal BP and pre-hypertension are presented in Table 1. The results displayed in Table 1 indicate that the pre-hypertension and normal BP groups were significantly different in terms of sex, age, BMI, and mean SBP and DBP.

During the 5-year follow up period, among the participants with normal BP, 119 (9.7%) developed type 2 diabetes, compared to 179 patients with pre-hypertension (12.7%). The unadjusted RR of developing diabetes for females with pre-hypertension compared to females with normal BP was 1.3 (95% confidence interval [CI], 1.0 to 1.7) and for males it was 1.6 (95% CI, 1.1 to 2.5), with no significant difference observed between the sexes.

After modeling the exposure to control for confounding factors, modeling the censored cases to adjust for selection bias, and calculating the weight of each individual through IPTW, the risk difference between the pre-hypertension and normal BP groups was calculated. The results of this analysis showed that the risk of diabetes incidence in the group with normal BP was 10.7%, while it was 12.1% in the group with pre-hypertension. The ATE was estimated to be 1.4% (95% CI, −1.1 to 3.4; p= 0.28). The RR for individuals with pre-hypertension was estimated to be 1.13 (95% CI, 0.90 to 1.41) (Table 2).

The participants were also analyzed based on the JNC-6 criteria for high-normal BP and normal BP, in comparison with optimal BP. The risk of developing diabetes in people with optimal BP was 9.7%, while it was 10.7% for those with normal BP and 15.3% for those with high-normal BP. The RRs of participants with normal BP and high-normal BP compared to those with optimal BP were, respectively, 0.96 (95% CI, 0.73 to 1.25) and 1.32 (95% CI, 1.01 to 1.72) (Table 2). According to our results, high-normal BP levels (130-139/85-89 mmHg) were a risk factor for developing diabetes mellitus.

## DISCUSSION

In this study, the unadjusted 5-year risk of diabetes among people with pre-hypertension was higher than that among those with normal BP. However, after modeling the exposure and controlling for covariates, pre-hypertension had no significant effect on the incidence of diabetes within the 5-year period. This is consistent with the findings of San Antonio study conducted by Mullican et al. [19]. In their study, they reported that BMI was the most important confounding factor. In our study, the risk of developing diabetes among participants with high-normal BP (120-139 mmHg and/or 85-89 mmHg) was higher than that among those with optimal BP, as in Mullican’s study (odds ratio, 1.69; 95% CI, 1.03 to 2.77) [19]; this range of BP significantly increased the risk of diabetes incidence. Despite the similar results, the 2 studies were different in terms of the length of the study (in Mullican’s study, the median follow-up length was 7.8 years, but in the current study it was 5 years), the age range of the participants (25 to 65 years of age in Mullican’s study), and the data analysis method [19]. Calculation of the diabetes incidence for different age groups in Mullican’s study showed that for participants younger than 50 years of age, the risk of diabetes incidence was significantly higher among people with pre-hypertension [19]. The different age ranges in the 2 studies can explain the stronger effect of high-normal BP on the incidence of diabetes in Mullican’s study than in our study. In a critical expert review of Mullican’s study, Everett & Frithsen [30] referred to the necessity of dividing pre-hypertension into 2 groups and of considering BP of 130-139 and/or 85-89 mmHg as a risk factor for type 2 diabetes. A study by Conen et al. [2] also emphasized that high-normal BP, based on the JNC-6 criteria, was a risk factor for females. Despite the non-significant effect of pre-hypertension on type 2 diabetes, some studies have emphasized the necessity of dividing pre-hypertension into 2 groups based on the JNC-6 criteria. Our results also showed that high-normal BP, compared with optimal BP, was associated with an increased risk of diabetes incidence, which is in line with the results of other similar studies [2,30,31]. In the new guideline, Whelton et al. [21] said “stage 1 hypertension is the appropriate term and that will capture the risk for adults and for clinicians much better”.

The difference in the method of data analysis is another factor that distinguishes our study from others. In the method used in this study, the inverse of pre-hypertension probability and normal BP in the levels of confounding variables were used to calculate the average weighted incidence of diabetes according to levels of exposure. The advantage of this method over regression methods is that it makes a causal interpretation possible. In many cases, covariate variables simultaneously act as confounders and effect modifiers. Traditional methods, such as regression, investigate the effect of exposure with the assumption of no interaction between the effects of the exposure and the confounding factors [32,33], but in our model we entered BMI and education years as continuous variables with an interaction term. We also entered age as a continuous and restricted cubic-spline variable.

One of the weaknesses of the current study is that patients with CVD were not excluded from the study, which is a potential limitation because of the association of CVD with the exposure and outcome of the study. Moreover, other factors associated with obesity, such as waist circumference, drinking, and physical activity were not measured in the first phase of the study. The strengths of this study include the modeling of the exposure and modeling of the censored participants in the second phase of the study based on confounding variables, such as BMI and age.

In summary, the results of this study showed that pre-hypertension, based on the JNC-7 definition, was not a risk factor for the incidence of diabetes. Our results showed that among the participants who had pre-hypertension, those with higher BP levels (high-normal compared to normal BP) had a higher risk of developing diabetes. These results show a positive relationship between increased BP and diabetes incidence risk among adults. Comparing these results with those other studies confirms that since BP measurement is a quantitative and continuous variable, the use of the new definition of stage 1 hypertension (according to ACA/ AHA guideline) may be a more meaningful categorization for diabetes risk assessment than the JNC-7 classification. Persons in the high-normal stage are already at a substantially increased risk of diabetes compared to those with normal BP. To reduce diabetes incidence in the adult population, controlling and monitoring BP is recommended.

## Figures and Tables

**Table 1.** Comparison of baseline characteristics among participants with normal BP and pre-hypertension

Characteristics	Normal BP (n=1,364)	Pre-hypertension (n=1,577)	Total (n=2,941)	p-value
Age (mean±SD, yr)	49.0±5.7	50.1±6.1	49.6±5.9	<0.001
BMI (mean±SD, kg/m^2^)	26.7±4.7	28.1±4.7	27.4±4.7	<0.001
Years of education (mean±SD, yr)	7.5±4.5	7.6±4.6	7.6±4.6	0.73
SBP (mean±SD, mmHg)	109.8±7.4	126.8±6.6	118.9±11.0	<0.001
DBP (mean±SD, mmHg)	69.4±6.5	79.3±6.3	74.7±8.1	<0.001
Sex				
Male	507 (37.2)	751 (47.6)	1,258 (42.8)	<0.001
Female	857 (62.8)	826 (52.4)	1,683 (57.2)	
BMI				
Normal	507 (7.2)	395 (25.1)	902 (30.7)	<0.001
Overweight	545 (40.0)	683 (43.3)	1,228 (41.8)	
Obese	312 (22.9)	499 (31.6)	811 (27.6)	
Tobacco smoking	205 (15.0)	197 (12.5)	402 (13.7)	0.05
Economic status				
High	643 (47.2)	748 (47.5)	1,391 (47.4)	0.97
Medium	378 (27.7)	437 (27.8)	815 (27.8)	
Low	342 (25.1)	389 (24.7)	731 (24.8)	

Values are presented as number (%). BP, blood pressure, SD, standard deviation; BMI, body mass index; SBP, systolic blood pressure; DBP, diastolic blood pressure.

**Table 2.** Risk of incident diabetes over 5 years in the adult population of Shahroud using inverse probability treatment weighting methods in a log binomial regression model

Classification	Definition (mmHg)	Risk ratio (95% CI)
JNC-7		
Normal	SBP <120 and DBP <80	1.00 (reference)
Pre-hypertension	SBP 120-139 and/ or DBP 80-89	1.13 (0.90, 1.41)
JNC-6		
Optimal	SBP <120 and DBP <80	1.00 (reference)
Normal	SBP 120-129 and/ or DBP 80-84	0.96 (0.73, 1.25)
High-normal	SBP 130-139 and/ or DBP 85-89	1.32 (1.01, 1.72)

CI, confidence interval; JNC, Joint National Committee on Prevention, Detection, Evaluation, and Treatment of High Blood Pressure; SBP, systolic blood pressure; DBP, diastolic blood pressure.
